# Chirality Inherent and Induced: An Algebraic Point of View

**DOI:** 10.1021/jacs.6c13874

**Published:** 2026-09-29

**Authors:** James R. Hamilton, Francoise Remacle, Raphael D. Levine

**Affiliations:** † Institute of Chemistry, 26742 The Hebrew University of Jerusalem, Jerusalem 91904, Israel; ‡ Theoretical Physical Chemistry, UR MOLSYS, 26658 University of Liege, Liège B4000, Belgium; § Department of Molecular and Medical Pharmacology, David Geffen School of Medicine, University of California, Los Angeles, California 90095, United States; ∥ Department of Chemistry and Biochemistry, University of California, Los Angeles, California 90095, United States

## Abstract

IUPAC defines chirality as a geometric property of an object. Here we discuss a complementary view of chirality in algebraic terms. This enables us to extend its definition from classical to quantum mechanics. We further infer that classical chirality can only be induced in linear combinations of coherences between quantum states. A current motivation for our approach is the ability to induce chirality in an achiral system by a chiral perturbation, most notably an ultrafast light pulse. The stereodynamics of the electrons will vary rapidly with time. Pumping the electronic states of the hydrogen atom provides an explicit example which is readily visualized. The algebraic definition allows a generalization from a classical, local, to a quantum mechanical, non-local concept of chirality.

## Graphical abstract

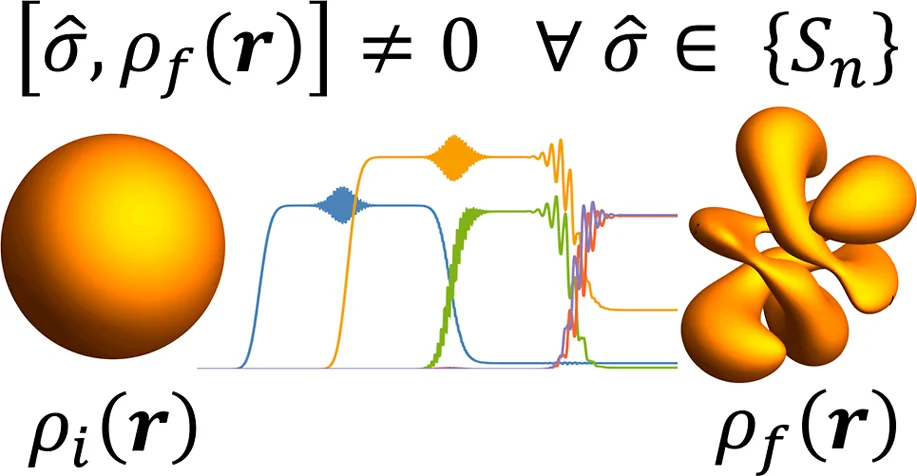

## Introduction

Chirality is an evergreen, perennially challenging and firing chemists’ intuition. A recent example is the chiral induced spin selectivity,^1^ which spawned important developments in allied areas.^2–9^ Other notable experiments in light-to-matter chirality transfer involve the formation of chiral nanoparticles from circularly polarised light.^10^ A threshold was crossed by the recognition that it is not necessary to regard chirality as a binary property. Rather, the chirality of an object is better conceived of as a continuous variable falling between the extremes of fully chiral and fully achiral. This led to a significant increase in publications. Already in 2003, a well annotated review^11^ of symmetry and chirality measures listed over 200 relevant papers. The celebrated overviews of continuous symmetry measures,^12,13^ (CSMs) provided a conceptual and a practical approach for a variety of general mathematical, but not necessarily geometric, objects. For quantum mechanical applications, more specific CSMs have been proposed for specific objects, such as the wavefunction.^14^

Important conceptual developments were made when it was understood that chirality is not limited to being a geometric property.^15^ In a subsequent publication, we will look at the time dependency of the probability densities introduced and discussed in the results section of this manuscript in order to explore the temporality of chirality. To do this, we will draw on Barron’s work and ideas, which show that chirality can be defined by non-invariance under time-reversal, in addition to non-inversion under geometric parity inversion.

An evergreen is an evergreen. The recent spectacular abilities in tailoring laser pulses, particularly very short ones, have spawned a new activity, that of inducing chirality.^6,16–18^ Specifically, one starts with an achiral initial state which is pumped by a chiral set of fs laser pulses.^19^

Chirality was first conceived of at the quantum level by Hund.^20,21^ When applying concepts such as symmetry and chirality in quantum mechanics, additional concepts such as non-locality need to be considered. Quantum non-locality is the coherence between the wavefunction at different points in coordinate space. A more rigorous mathematical definition will be given below, but it is easily understood that the phenomena is due to the wave nature of matter and so is more important for lighter particles and electrons in particular. This paper concerns itself primarily with chirality as it is more commonly conceived of, that is as a geometric property of the local density. In this sense, a system is chiral when it cannot be superimposed onto any of its enantiomers (its images after being acted upon by operators in the sets of symmetry operations which define chirality, for example reflection in any of the three Cartesian planes). It is possible and in certain cases insightful to discuss the chiral aspects of the non-local density because it is a direct manifestation of quantum mechanics. While we will emphasize the much more common local aspects we do provide below simple illustrations of the non-locality.

### The Group Theory Definition of Chirality

In mechanics we need to specify the equations of motion and, quite independently, the initial conditions needed to solve these equations. In this paper we use an algebraic approach and so we require the algebra that expresses the symmetry of the initial (or final) state and the algebra that defines the dynamics. The former is known for some time. The compact way is to use the orthogonal group O(3) in three dimensions. The action of an element *R̂* of the group leaves the origin fixed and preserves the Euclidean distance, $\mathrm{R̂}{\mathrm{R̂}}^{T}=Î$ where ${\mathrm{R̂}}^{T}$ is the transpose and *Î* is the identity operator. The O(3) group has two components: proper rotations for which the determinant of ${\mathrm{R̂}}^{P}$ is unity; and improper rotations, which are a product of proper rotations and perpendicular reflections, for which the determinant of ${\mathrm{R̂}}^{I}$ is −1.

At the risk of being pedantic, a Lie group needs to allow transformations very near the identity, thereby enabling continuous transformations. The pure rotations commute and close a subgroup, a Lie group SO(3). Discrete operations are definable from the continuous operators. The discrete, *n*th order proper rotation operators constitute a symmetry group, $C_{n}=\left\{{\mathrm{R̂}}_{x}^{P}\left(n\right),{\mathrm{R̂}}_{y}^{P}\left(n\right),{\mathrm{R̂}}_{z}^{P}\left(n\right)\right\}$ . The operators of this group rotate a vector or function by 2π/*n* radians around the *x*,*y* and *z* axes. An improper rotation operator is the product of a proper rotation and an inversion, *S*_*n*_ = σ_*h*_ *C*_*n*_, where σ_*h*_ is a horizontal or perpendicular reflection. In this way, the discrete *n*th order improper rotation operators constitute a symmetry, $S_{n}=\left\{{\mathrm{R̂}}_{x}^{I}\left(n\right),{\mathrm{R̂}}_{y}^{I}\left(n\right),{\mathrm{R̂}}_{z}^{I}\left(n\right)\right\}$ . The constituent operators of *S*_*n*_ rotate a vector or function by 2π/*n* radians around the specified axis and then reflect it in the plane perpendicular to this axis of rotation. More explicitly, one can write ${\mathrm{R̂}}_{i}^{I}\left(n\right)={\mathrm{\sigma ̂}}_{j,k}{\mathrm{R̂}}_{i}^{P}\left(n\right)$ where *i* ≠ *j*,*k*. ${\mathrm{\sigma ̂}}_{j,k}$ is the reflection operator of the *j*–*k* plane, acting as, for example, ${\mathrm{\sigma ̂}}_{xy}f\left(x,y,z\right)=f\left(x,y,-z\right)$ and so on. Note that, since ${\mathrm{R̂}}_{i}^{P}\left(1\right)$ is just a rotation by 2π around the axis *i*, and hence ${\mathrm{R̂}}_{i}^{P}\left(1\right)=Î\;\forall \;i$ , therefore the first order improper rotations are just the reflection operators $S_{1}=\left\{{\mathrm{\sigma ̂}}_{xy},{\mathrm{\sigma ̂}}_{xz},{\mathrm{\sigma ̂}}_{yz}\right\}$ . It is also interesting to note that

$${\mathrm{R̂}}_{x}^{I}\left(2\right)f\left(x,y,z\right)={\mathrm{R̂}}_{y}^{I}\left(2\right)f\left(x,y,z\right)={\mathrm{R̂}}_{z}^{I}\left(2\right)f\left(x,y,z\right)=f\left(-x,-y,-z\right)$$

and therefore the second order symmetry group only contains a single operator $S_{2}=\left\{{\mathrm{R̂}}^{I}\left(2\right)\right\}.$ By its action, it is clear that this operator is the physical parity operator, although we will continue to use the symbol for improper rotation. The improper rotations are a component of the Lie group O(3), but, as they are not closed under commutation, do not themselves form a Lie group.

The classical chirality of atoms and molecules is a geometric property of their density function in physical coordinate space ρ­(**r**). A locally defined density function is chiral if it is non-invariant under the full set of improper symmetry operations, of all orders

1 $$S_{n}\rho \left(r\right)\neq \rho \left(r\right)\;\text{for}\;\text{all}\;n$$

As is usefully recognized, almost all achiral molecules, about 90%, are invariant already for *n* = 1. The next 9% will be invariant for *n* = 2, a significantly smaller percentage will be invariant for *n* = 4 etcetera (ref 13 and references therein).

It is later shown that this group theory definition of chirality is derivable from the algebraic definition. As the group and algebraic definitions of chirality build upon the definitions of symmetry. It is therefore useful to precede a discussion of chiral atomic densities with an example of an asymmetric atomic density which is non-invariant under a particular reflection, while remaining invariant under the other two.

### From Groups to Algebras

Lie groups^22^ deal with continuous symmetries, which include infinitesimal changes near the identity. As discussed in introductory texts, it is thereby possible to introduce the corresponding algebras whose elements are the generators of the group. This is achieved by an exponential map between the elements of the algebra and those of the group. In physical, three-dimensional space, the familiar angular momentum operator, ${Ĵ}_{j}\equiv -i\partial /\partial \vartheta$ is the generator of a finite rotation by angle Δϑ about the axis *j*, ${\mathrm{R̂}}_{j}\left(\Delta \vartheta \right)=\exp\left(-i\Delta \vartheta {Ĵ}_{j}\right)$ . As we show in Section S1 of the Supporting Information, ${\mathrm{R̂}}_{j}\left(\Delta \vartheta \right)$ is a Taylor operator, meaning that it advances the argument of a function *f*(ϑ) by Δϑ, ${\mathrm{R̂}}_{j}\left(\Delta \vartheta \right)f\left(\vartheta \right)=f\left(\vartheta -\Delta \vartheta \right)$ .

The elements of the group need not commute, but their product needs to be a group element. It follows from the first term in the Zassenhaus formula that the set of corresponding generators must be closed under commutation. For example, ${Ĵ}_{x}{Ĵ}_{y}-{Ĵ}_{y}{Ĵ}_{x}\equiv \left[{Ĵ}_{x},{Ĵ}_{y}\right]=i{Ĵ}_{z}$ , and similarly for the other two directions.

In 3-dimensional Cartesian space the elements of the SO(3) rotation group have matrix representations. For example

$$R_{z}\left(\Delta \vartheta \right)=\left(\begin{array}{lll} \cos\left(\Delta \vartheta \right) & \sin\left(\Delta \vartheta \right) & 0\\ -\sin\left(\Delta \vartheta \right) & \cos\left(\Delta \vartheta \right) & 0\\ 0 & 0 & 1 \end{array}\right)$$

rotates a vector (*x*,*y*,*z*)^*T*^ by angle Δϑ about the *z* axis. This is a continuous rotation, ${\mathrm{R̂}}_{z}\left(\Delta \vartheta \right)=\exp\left(-i\Delta \vartheta {Ĵ}_{z}\right)$ , from which the discrete proper rotation is defined ${\mathrm{R̂}}_{z}\left(n\right)=\exp\left(-i\left(2\pi /n\right){Ĵ}_{z}\right)$ . Δϑ is a finite but continuous angle, whereas *n* is a discrete integer. Similarly, from the continuous rotations ${\mathrm{R̂}}_{x}\left(\Delta \vartheta \right)=\exp\left(-i\Delta \vartheta {Ĵ}_{x}\right)$ and ${\mathrm{R̂}}_{y}\left(\Delta \vartheta \right)=\exp\left(-i\Delta \vartheta {Ĵ}_{y}\right)$ one can define the discrete proper rotations ${\mathrm{R̂}}_{x}\left(n\right)=\exp\left(-i\left(2\pi /n\right){Ĵ}_{x}\right)$ and ${\mathrm{R̂}}_{y}\left(n\right)=\exp\left(-i\left(2\pi /n\right){Ĵ}_{y}\right)$ . The set of discrete proper rotations $\left\{{\mathrm{R̂}}_{x}\left(n\right),{\mathrm{R̂}}_{y}\left(n\right),{\mathrm{R̂}}_{z}\left(n\right)\right\}$ constitutes *C*_*n*_.

Consider a reflection operator in a plane, say the *x*–*z* plane. In Cartesian coordinates this operator acts as, ${\mathrm{\sigma ̂}}_{xz}f\left(x,y,z\right)=f\left(x,-y,z\right)$ , or in spherical polar coordinates as ${\mathrm{\sigma ̂}}_{xz}f\left(r,\theta ,\phi \right)=f\left(r,\theta ,-\phi \right)$ . In general conceptualization and usage, the reflections are a discrete symmetry. The matrix

$$\sigma_{xz}=\left(\begin{array}{lll} 1 & 0 & 0\\ 0 & -1 & 0\\ 0 & 0 & 1 \end{array}\right)$$

reflects a vector in the *x*–*z* plane of Cartesian space, with equivalent expressions for **σ**_*xy*_ and **σ**_*yz*_.

The action of these reflections is not near an identity, so they do not close a Lie group and, ordinarily, one does not associate a generator with them. However, one can see this reflection as a discrete instance of an abstract, continuous operator for a plane, say the *x*–*z* plane.

2 $${\mathrm{\sigma ̂}}_{xz}\left(\Delta \vartheta \right)=\exp\left[-i\Delta \vartheta {\mathrm{\varpi ̂}}_{xz}\right]$$

The continuous operator ${\mathrm{\sigma ̂}}_{xz}\left(\Delta \vartheta \right)$ “reflects” a geometric function by Δϑ along the *y*-axis perpendicular to the *x*–*z* plane. The regular reflection is then a reflection by Δϑ = π. The “reflections” produced by the action of the continuous operator ${\mathrm{\sigma ̂}}_{xz}\left(\Delta \vartheta \right)$ are akin to the distortions one sees in carnival mirrors.

$$\sigma_{xz}\left(\Delta \vartheta \right)=\left(\begin{array}{lll} 1 & 0 & 0\\ 0 & \exp\left[i\Delta \vartheta \right] & 0\\ 0 & 0 & 1 \end{array}\right)$$

Unlike the funfair mirrors, however ${\mathrm{\sigma ̂}}_{xy}\left(\Delta \vartheta \right)$ stretches a function into both real and imaginary components. It scales the angle ϕ all the way to -ϕ (when Δϑ = π) and beyond. The generator of reflection in the *x*–*z* plane is ${\mathrm{\varpi ̂}}_{xz}\equiv \phi \left(\partial /\partial \phi \right)$ . The proof of this statement is provided in Section S2 of the Supporting Information. Therefore, eq 2 becomes ${\mathrm{\sigma ̂}}_{xz}\left(\Delta \vartheta \right)=\exp\left[-i\Delta \vartheta \phi \left(\partial /\partial \phi \right)\right]$ . What this implies is that it scales the variable ϕ to exp­(−*i*Δϑ)­ϕ, see Section S2 of the Supporting Information. This is analogous to the complex scaling used in quantum chemistry, particularly in the theory of resonances.^23,24^

In the scaling notation, the conventional reflections which, along with the proper rotations, constitute the sets of improper rotations *S*_*n*_ are σ_*xy*_(Δϑ = π), σ_*xz*_(Δϑ = π) and σ_*yz*_(Δϑ = π). However, as this is the only kind of reflection we use in this particular work, the explicit specification of Δϑ = π will be dropped. As is conventional, we will write ${\mathrm{\sigma ̂}}_{xy}$ , ${\mathrm{\sigma ̂}}_{xz}$ and ${\mathrm{\sigma ̂}}_{yz}$ .

We now make an intermezzo to illustrate an example of a continuous reflection transformation. We will plot below the results for a state that is a superposition of a 3p, *m* = 1 and a 3d, *m* = −2 atomic orbitals of hydrogen. As will be shown both analytically and numerically this results in a three-fold symmetry. This state is not an eigenstate of the orbital angular momentum or of its projection *m*. States of common quantum number *n* are degenerate but for non-degenerate states the probability density will exhibit precession around the *z*-axis. With a choice of a (3d, *m* = 2) state the angular part will look quite different. Our two test states are

3 $$\psi_{\pm }\left(r,\theta ,\phi \right)=\frac{1}{\sqrt{2}}\left(\psi_{3,1,1}\left(r,\theta ,\phi \right)+\psi_{3,2,\pm 2}\left(r,\theta ,\phi \right)\right)$$

where the subscripts on the hydrogenic orbitals are *n*,*l*,*m*, the familiar principal, angular and azimuthal quantum numbers. We use the standard factorization ψ_*n*,*l*,*m*_(*r*,θ,ϕ) = *R*_*n*_^*l*^(*r*)­Θ_*l*_^*m*^(θ)​exp­(*im*ϕ). In the *x*–*y* plane, where θ = π/2, the angular properly normalized probability densities are *P*_+_(θ = π/2,ϕ) = (2 + 2 cos­(ϕ))/4π,*P*_–_(θ = π/2,ϕ) = (2 + 2 cos­(3ϕ))/4π. The complete probability density in the *x*–*y* plane is ${\left|\psi_{-}\left(r,\theta =\pi /2,\phi \right)\right|}^{2}$ and this density also has radial dependence.

We next illustrate the scaling, using the explicit result exp­(−*i*Δϑϕ∂/∂ϕ) *f*(θ,ϕ) = *f*(θ,exp­(−*i*Δϑ)­ϕ). So upon scaling ϕ → ϕ_*r*_ – *i*γ,ϕ_*r*_ = ϕ cos­(Δϑ),γ = ϕ sin­(Δϑ), where γ is the imaginary part of ϕ that is due to the complex scaling. Substituting into the angular components: 3p state: exp­(*i*ϕ) → exp*i*(ϕ_*r*_ – *i*γ) = exp­(γ)­exp­(*i*ϕ_*r*_), while for the 3d, *m* = 2 state: exp­(–2*i*ϕ) →exp­(–2*i*(ϕ_*r*_–*i*γ)) = exp­(–2γ)­exp­(–2*i*ϕ_*r*_). Note how the 3d, *m* = 2 state scales quite differently from the 3d, *m* = −2 state. Then

$$P_{+}\left(\theta =\pi /2,\phi \right)\propto \exp\left(-2\gamma \right)+\exp\left(-4\gamma \right)+2\exp\left(-3\gamma \right)\cos\left(\phi_{r}\right)$$

and

$$P_{-}\left(\theta =\pi /2,\phi \right)\propto \exp\left(2\gamma \right)+\exp\left(-4\gamma \right)+2\exp\left(-\gamma \right)\cos\left(3\phi_{r}\right)$$

where we are varying Δϑ and changing both ϕ_*r*_ and γ accordingly. The purpose of the continuous scaling is to allow changes near the identity, so we take few small values of Δϑ. Figure 1 is a plot of the two resulting functions above. It is no longer a probability density because to normalize the probability to unity we need to also scale the area element in the *x*–*y* plane.

**Figure 1:**
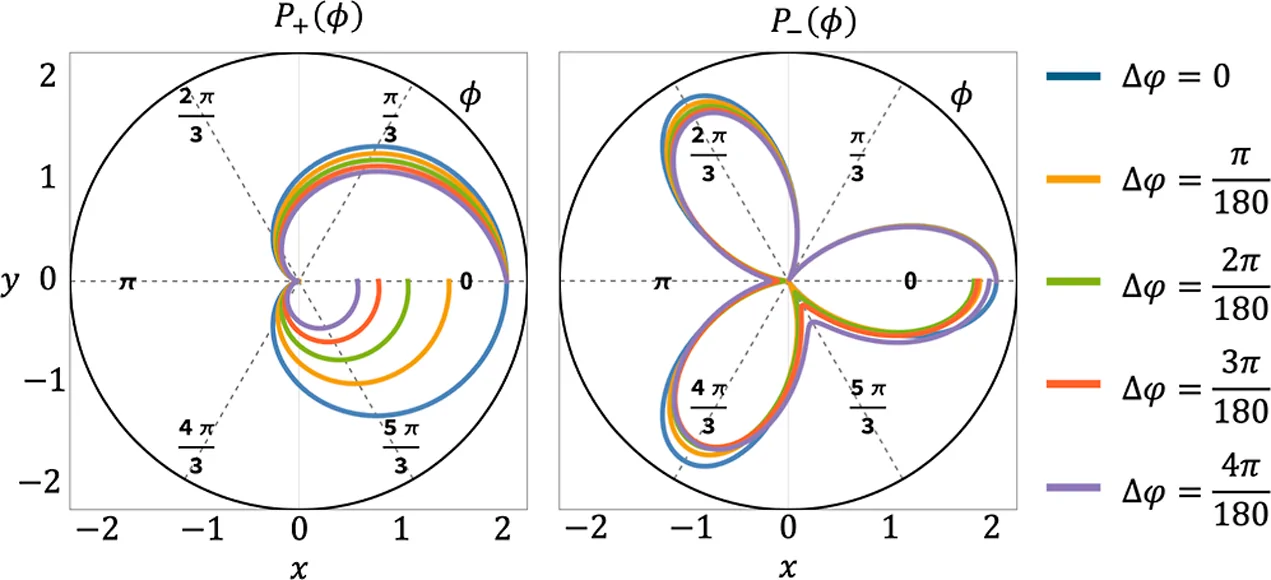
Argand diagram plots of the (unnormalized) probability densities upon scaling near the identity for two similar wave functions shown analytically as eq 3. These are linear combinations of a hydrogenic 3p, *m* = 1 orbital and a 3d, *m* = ±2 orbital. The angle ϕ varies in a counterclockwise manner (starting from ϕ = 0 at 3 o’clock). To make it rotate in the opposite manner rotates the panels along the *y* axis. The normalized densities need to be divided by a γ dependent constant and will have a unit area under them but their shapes will look the same. The increments in Δϑ, the angle of scaling, shown on the right, are in radians.

### The Algebraic and Group Definitions of Chirality

Textbooks of quantum mechanics and/or symmetry in chemistry show that a system is not spherically symmetric if its state, ρ­(***r***), fails to commute with the operators of the angular momentum, $\left[{Ĵ}_{i},\rho \left(r\right)\right]\neq 0$ . Similarly, we will take the algebraic definition of chirality of a quantum mechanical state to be its failure to commute with the generators of the *S*_*n*_, $\left[{\mathrm{\varpi ̂}}_{ij},\rho \left(r\right)\right]\neq 0\;and\left[{Ĵ}_{j}\left(n\right),\rho \left(r\right)\right]\neq 0$ , where ${Ĵ}_{i}\left(n\right)$ are the generators of the discrete improper rotations, ${\mathrm{R̂}}_{j}^{I}\left(n\right)=\exp\left[-i\left(2\pi /n\right){Ĵ}_{j}\right]$ .

The group definition of chirality in terms of the operators, eq 1, follows from the algebraic definition in terms of the generators. Using a generic operator exponentially mapped to its corresponding generator X̂=exp[Ŷ]

$$\left[\mathrm{X̂},\rho \left(r\right)\right]=\left[\exp\left[Ŷ\right],\rho \left(r\right)\right]=\left[\sum_{k=0}^{\infty }{Ŷ}^{k},\rho \left(r\right)\right]=\sum_{k=0}^{\infty }\left[{Ŷ}^{k},\rho \left(r\right)\right]$$

Therefore, if the density commutes with the generators of the algebra, it likewise commutes with the operators of the group.

We emphasize that we use this definition for both the full non-local quantum density matrix, ρ­(**r**′;**r**) ≡ ⟨**r**′|**ρ**|**r**⟩, and for the local, geometric density, ρ(r)≡⟨r|ρ̂|r⟩ This latter is the diagonal element of the density operator in the coordinate representation, and the former are the off-diagonal elements.

It is worth making a point of clarification here. In much of this manuscript, we will analyze the asymmetry and chirality of the local, geometric density ρ­(**r**). This being a quantum mechanical probability density, this chirality is, in a sense, quantum mechanical. However, when we refer to quantum chirality in this work, we do not mean the chirality of quantum objects. Rather, we are referring to the chirality of non-localised forms of these objects, such as the coherences between two different points in geometric space in the coordinate representation of the density operator, ρ­(**r**′;**r**).

The definition of quantum mechanical chirality, the chirality of a non-local coherence ρ­(**r**′,**r**), is the same as that of a local density, ρ­(**r**). The group definition is that ρ­(**r**′,**r**) has quantum chirality if it is non-invariant under the operations of all improper rotations. The algebraic definition is its non-commutation with the generators of all improper rotations.

As an example of the action of an *S*_*n*_ symmetry operation, let us take the S_1_ reflection in the *x*–*y* plane, ${\mathrm{\sigma ̂}}_{xy}\rho \left(x^{\prime },y^{\prime },z^{\prime };x,y,z\right)=\rho \left(x^{\prime },y^{\prime },-z^{\prime };x,y,-z\right)$ . It follows that the action of the commutator $\left[{\mathrm{\sigma ̂}}_{xy},\rho \left(r^{\prime };r\right)\right]$ on a test function *f*(**r**′;**r**) is

4 $$\begin{array}{l} \left[{\mathrm{\sigma ̂}}_{xy},\rho \left(r^{\prime };r\right)\right]f\left(r^{\prime };r\right)\\ =\rho \left(x^{\prime },y^{\prime },-z^{\prime };x,y,-z\right)f\left(x^{\prime },y^{\prime },-z^{\prime };x,y,-z\right)\\ -\rho \left(x^{\prime },y^{\prime },z^{\prime };x,y,z\right)f\left(x^{\prime },y^{\prime },-z^{\prime };x,y,-z\right)\\ =\left\{\rho \left(x^{\prime },y^{\prime },-z^{\prime };x,y,-z\right)-\rho \left(x^{\prime },y^{\prime },z^{\prime };x,y,z\right)\right\}f\left(x^{\prime },y^{\prime },-z^{\prime };x,y,-z\right) \end{array}$$

Since we defined ρ(r)≡⟨r|ρ̂|r⟩ we have that for the density

$$\left[{\mathrm{\sigma ̂}}_{xy},\rho \left(x,y,z\right)\right]f\left(x,y,z\right)=\rho \left(x,y,-z\right)f\left(x,y,-z\right)-\rho \left(x,y,z\right)f\left(x,y,-z\right)=\left\{\rho \left(x,y,-z\right)-\rho \left(x,y,z\right)\right\}f\left(x,y,-z\right)$$

For the density we recover the familiar result: a density is invariant, $\left[{\mathrm{\sigma ̂}}_{xy},\rho \left(r\right)\right]=0$ if it is identical to its enantiomer, produced by reflection in the *x*–*y* plane

ρ(x,y,−z)=ρ(x,y,z)

Similarly for a reflection in the other two planes.

For a general quantum state, it is quantum mechanically symmetric if also the coherence is invariant

$$\rho \left(x^{\prime },y^{\prime },-z^{\prime };x,y,-z\right)=\rho \left(x^{\prime },y^{\prime },z^{\prime };x,y,z\right)$$

The group definition of chirality follows from this definition of asymmetry: A density is chiral if it fails to commute with all the reflections and improper rotations of *S*_*n*_ ∀ *n*. A general state is chiral if also its coherences fail to be invariant. As has been said, most achiral objects are invariant in S_1_, S_2_ and/or S_4_ (ref 13and references therein). In practice, therefore, to test the chirality of a density, it is sufficient only to test it up to S_4_.

This yields an explicit expression for all of the commutators corresponding to the operators of all the *S*_*n*_. For example, for the density and the reflection in the *x*–*y* plane

$$\left[{\mathrm{\sigma ̂}}_{xy},\rho \left(r\right)\right]=\left\{\rho \left(x,y,-z\right)-\rho \left(x,y,z\right)\right\}{\mathrm{\sigma ̂}}_{xy}$$

The result of the failure to commute produces a coefficient, {ρ­(*x*,*y*,–*z*)–ρ­(*x*,*y*,*z*)},times an operator that is in the group O(3).

The equation ρ­(*x*,*y*,–*z*) = ρ­(*x*,*y*,*z*) is equivalent to ${\mathrm{\sigma ̂}}_{xy}\rho \left(x,y,z\right)=\rho \left(x,y,z\right)$ . Therefore, the group definition of chirality, eq 1, follows from the algebraic definition, $\left[{\mathrm{\sigma ̂}}_{xy},\rho \left(r\right)\right]=0$ . The magnitude of the deviation, {ρ­(*x*,*y*,–*z*)–ρ­(*x*,*y*,*z*)}­is a measure of the chirality of the density or, in the general quantum state, a measure of the chirality of the coherence. This measure of chirality is the *odd* part of the density.

We illustrate the difference in the density and its enantiomer for two different reflections in the polar coordinates ϕ and θ using a wave function that is a coherent superposition of two hydrogenic orbitals $\psi \left(r\right)=\left(\psi_{3,1,1}\left(r\right)+\psi_{3,2,1}\left(r\right)\right)/\sqrt{2}$ The delocalized density is defined from this wavefunction as

5 $$\rho \left(r^{\prime },r\right)\equiv \psi^{*}\left(r^{\prime }\right)\psi \left(r\right)$$

This delocalized density will be reflected in the *x*–*z* plane, which transformation only affects the azimuthal part, ${\mathrm{\sigma ̂}}_{xz}f\left(r,\theta ,\phi \right)=f\left(r,\theta ,-\phi \right)$ , and, separately, also in the *x*–*y* plane, which only affects the angular part, ${\mathrm{\sigma ̂}}_{xy}f\left(r,\theta ,\phi \right)=f\left(r,\pi -\theta ,\phi \right)$ . Therefore, to examine the asymmetry in reflection in the *x*–*z* plane, the azimuthal part of the density is spotlit by taking *r*′ = *r* and θ′ = θ and integrating over these radial and angular coordinates

6 $$\rho \left(\phi^{\prime },\phi \right)\equiv \int_{0}^{\infty }\int_{0}^{\pi }\psi^{*}\left(r,\theta ,\phi^{\prime }\right)\psi \left(r,\theta ,\phi \right)r^{2}\sin\left[\theta \right]drd\theta =e^{i\left(\phi -\phi^{\prime }\right)}/2\pi$$

To focus on the asymmetry, we plot the measure that is the difference between the delocalized density and its enantiomer

7 $$Im\left[\rho \left(\phi^{\prime },\phi \right)-{\mathrm{\sigma ̂}}_{xz}\rho \left(\phi^{\prime },\phi \right)\right]=\mathrm{Sin}\left[\phi -\phi^{\prime }\right]/\pi$$

The result is shown as panel a of Figure 2, from ϕ′,ϕ = 0 – π.

**Figure 2:**
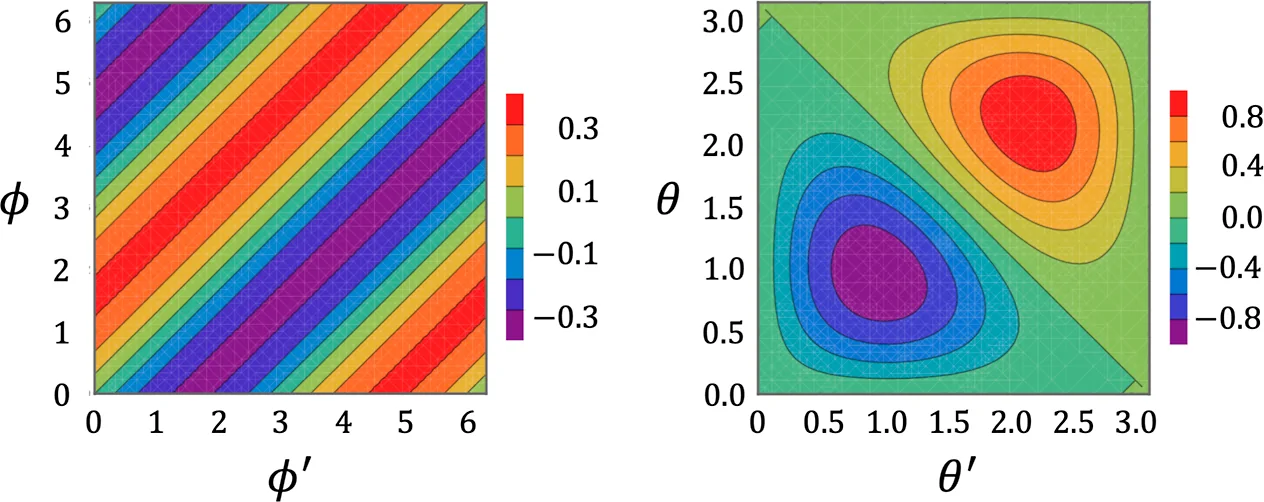
Plots showing the asymmetry of the delocalized density of the coherent superposition of two hydrogenic orbitals. Panel a shows the asymmetry of the reflection of this density in the *x*–*z* plane. The color bar on panel a shows the value of eq 7, the difference between the delocalized density and its enantiomer in the *x*–*z* plane, evaluated at the coordinates the (ϕ′,ϕ). This is a unitless quantity. Panel b shows the asymmetry of its reflection in the *x*–*y* plane. The color bar on panel b shows the value of eq 8, the difference between the delocalized density and its enantiomer in the *x*–*y* plane, evaluated at the coordinates (θ′,θ). The angles are shown in radians.

To examine the asymmetry under reflection in the *x*–*y* plane, the angular part is isolated by taking *r*′ = *r* and ϕ′ = ϕ and integrating these radial and azimuthal parts. The integral

$$\rho \left(\theta^{\prime },\theta \right)\equiv \int_{0}^{\infty }\int_{0}^{2\pi }\psi^{*}\left(r,\theta^{\prime },\phi \right)\psi \left(r,\theta ,\phi \right)r^{2}drd\phi =\frac{1}{8}\left(3-5\;\mathrm{Cos}\left[\theta^{\prime }\right]+5\;\mathrm{Cos}\left[\theta \right]\left(3\;\mathrm{Cos}\left[\theta^{\prime }\right]-1\right)\right)\mathrm{Sin}\left[\theta \right]\mathrm{Sin}\left[\theta^{\prime }\right]$$

and our measure that is the difference between the delocalized density and its enantiomer works out to

8 $$\rho \left(\theta^{\prime },\theta \right)-{\mathrm{\sigma ̂}}_{xy}\rho \left(\theta^{\prime },\theta \right)=-\frac{5}{4}\left(\mathrm{Cos}\left(\theta \right)+\mathrm{Cos}\left(\theta^{\prime }\right)\right)\mathrm{Sin}\left(\theta \right)\mathrm{Sin}\left(\theta^{\prime }\right)$$

This function is plotted from θ′,θ = 0 – π, and the result is shown as panel b of Figure 2.

### Where Does the Chirality Reside?

We discuss who or what can be chiral. A pure state of definite angular momentum cannot be chiral, neither can a mixture of such states. A chiral state and specifically a chiral state in the classical, local meaning must include terms that are off diagonal in an angular momentum representation. We begin with a more abstract brief introduction and then rederive these general conclusions for a general state of the hydrogen atom because the shapes of the atomic orbitals are familiar.

If we give the operator for a general state, see, for example^25^

9 $$\mathrm{\rho ̂}=\sum_{J,M;J^{\prime },M^{\prime }}|J^{\prime },M^{\prime }\rangle \langle J^{\prime },M^{\prime }\left|\mathrm{\rho ̂}|J,M\rangle \langle J,M\right|$$

The state has a diagonal part that is necessarily achiral because the states |*J*,*M*⟩⟨*J*,*M*| are invariant under rotations. Any chirality of this state must be due to the off-diagonal terms, the coherent terms of the state specified by ρ̂. Each such off-diagonal term has a part that is local $\langle r\left|J^{\prime },M^{\prime }\rangle \langle J^{\prime },M^{\prime }\right|\mathrm{\rho ̂}\left|J,M\rangle \langle J,M\right|r$ ⟩ which contributes to the classical density ⟨r|ρ̂|r⟩ . In the quantum world there is also the non-local density whose transformations depend on the symmetries of ⟨**r**′|*J*′,*M*′⟩⟨*J*,*M*|**r**⟩ when both **r**′and **r** are reflected or subject to improper rotation.

However, while specific coherence terms in the density can be asymmetric, that is non-invariant, under specific reflection operations, no specific coherences between states of the hydrogen atom are chiral. Chirality can only be induced in linear combinations of hydrogenic coherences.

Starting from an electronic wave function that is a linear combination of electronic states of the Hydrogen atom $\psi \left(r\right)=\sum_{n,l,m}c_{l,m}^{n}\left(t\right)\psi_{n,l,m}\left(r\right)$ with complex time dependent coefficients $c_{l,m}^{n}\left(t\right)\in C$ . The corresponding classical local density is

$${\left|\psi \left(r\right)\right|}^{2}=\sum_{n^{\prime },l^{\prime },m^{\prime }}{\left(c_{l^{\prime },m^{\prime }}^{n^{\prime }}\left(t\right)\psi_{n^{\prime },l^{\prime },m^{\prime }}\left(r\right)\right)}^{*}\sum_{n,l,m}c_{l,m}^{n}\left(t\right)\psi_{n,l,m}\left(r\right)$$

For our purpose this density can be written as a sum of two quite different terms

10 $${\left|\psi \left(r\right)\right|}^{2}=\sum_{n,l,m}{\left|c_{l,m}^{n}\left(t\right)\right|}^{2}{\left|\psi_{n,l,m}\left(r\right)\right|}^{2}+\sum_{\left(n^{\prime },l^{\prime },m^{\prime }\right)\neq \left(n,l,m\right)}{c_{l^{\prime },m^{\prime }}^{n^{\prime }}}^{*}\left(t\right)c_{l,m}^{n}\left(t\right){{\psi_{n^{\prime },l^{\prime },m^{\prime }}}^{*}\left(r\right)\psi }_{n,l,m}\left(r\right)$$

The first summation is over the population terms and does not contribute to the chirality. One can write explicit wave functions to show this invariance but familiarity with the orbital shapes of the hydrogen atom is probably sufficient to confirm this. If the overall density is chiral, it can only be because of the symmetry properties of the second summation, the linear combination of the coherences between different angular components. To be clear, the components of this summation are achiral, any coherence between two states of the hydrogen atom ${\psi_{n^{\prime },l^{\prime },m^{\prime }}}^{*}\left(r\right)\psi_{n,l,m}\left(r\right)$ will have at least one invariant symmetry. It is only in the linear combination of coherences that one can induce chirality.

### Chiral Dynamics

To pump a coherent superposition of states of different angular parts, states that will have densities that are not invariant under at least one order, *n*, of improper rotation, {*S*_*n*_}, it is possible to use two or more light pulses that are not collinear. An early study of interference of sigma and pi electronic states in a LiH diatomic molecule is.^26,27^ A more recent example is a study of NaK.^28^ To imprint chirality on an initially achiral state one needs a chiral laser pulse.^19^ An extensive discussion was provided of what this may create in a Hydrogen atom^6,16^ including explicit images of chiral electronic states of the atom. In this paper we discuss the actual dynamics of such pumping from an algebraic perspective.

The dynamical calculations are done in the Hilbert space spanned by the states of the hydrogen atom, {|*n*,*l*,*m*⟩}, where |*n*,*l*,*m*⟩ = |*nl*_*m*_⟩ signifies the hydrogenic state with principal quantum number *n*, angular momentum *l* and magnetic quantum number *m*. The time dependent Hamiltonian matrix of the pumped hydrogen atom is **H**(*t*) = **H**_0_ + **V**(*t*) where **H**_0_ is the Hamiltonian of the unperturbed Hydrogen atom, that is diagonal in the basis of states and where all the states of a given *n* quantum number are degenerate

$${H_{0}}_{n^{\prime },l^{\prime },m^{\prime };n,l,m}=\left\langle n^{\prime },l^{\prime },m^{\prime }|H_{0}|n,l,m\right\rangle =\{\begin{array}{l} E_{n}\\ 0 \end{array}\begin{array}{l} \text{if}\left(n^{\prime },l^{\prime },m^{\prime }\right)=\left(n,l,m\right)\\ \text{else} \end{array}$$

The dipole coupling to the time dependent field *E⃗*(*t*) with components along the three axes is

$$V\left(t\right)=-q\mathrm{E⃗}\left(t\right)\times \mathrm{r⃗}=-q\left(xE_{x}\left(t\right)+yE_{y}\left(t\right)+zE_{z}\left(t\right)\right)$$

We work in atomic units so that the charge on the electron, *q* = *e* = 1 and ℏ = 1. **V**(*t*) is the matrix representation of *V*(*t*), and the coordinates *x*, *y* and *z* are represented by the dipole matrices **X**, **Y** and **Z**. For example, $X_{n^{\prime },l^{\prime },m^{\prime };n,l,m}=\left\langle n^{\prime },l^{\prime },m^{\prime }|x|n,l,m\right\rangle$ These matrices define the well-known dipole selection rules, for example for the field along the *z*-axis with a resonance frequency, ω = *E*_*n*+1_ – *E*_*n*_, the allowed transition is |*n*,*l*,*m*⟩ → |*n* + 1,*l* ± 1,*m*⟩. The dipole matrices are rather sparse. Matrix plot representations of the **Z** and **X** matrices are shown in Section S3 of the Supporting Information. In Hilbert space, the perturbation is

11 $$V\left(t\right)=-E_{x}\left(t\right)X-E_{y}\left(t\right)Y-E_{z}\left(t\right)Z$$

The system evolved numerically using the Liouville von-Neumann equation

12 $$\frac{\partial \rho \left(t\right)}{\partial t}=-i\left[H\left(t\right),\rho \left(t\right)\right]$$

The numerical integrations for the dynamics are done by a Runge-Kutta algorithm written in Fortran and the density isosurface figures presented in this work were made in Mathematica^29^ using its built-in visualisation tools. Our codes and Mathematica notebooks are available on request.

### An Algebra for the Dynamics

For practical necessity we work in a Hilbert space of finite dimension, *N*. Therefore, all matrices that represent observables are *N* by *N* Hermitian matrices. All such matrices belong to the well-studied Lie algebra *U*(*N*). The most general basis set is the *N* ^2^ Gelfand matrices, {**E**_*n*,*m*_}, but smaller basis sets may be constructed which take advantage of the structure of the Hamiltonian. The matrix **E**_*n*,*m*_ has the entry unity at position *n, m* and zeros elsewhere. These matrices close the *U*(*N*) algebra, [**E**_*n*,*m*_,**E**_*i*,*j*_] = **E**_*n*,*j*_δ_*m*,*i*_ – **E**_*i*,*m*_δ_*j*,*n*_. The matrices **E**_*i*,*j*_ are the representation of the operators ${Ê}_{i,j}=\left|i\rangle \langle j\right|$ where for the Hydrogen atom the explicit result is |*i*⟩⟨*j*| = |*n*′,*l*′,*m*′⟩⟨*n*,*l*,*m*|. We have previously used these matrices extensively because, as will also be seen below, they enable a closed algebraic solution whenever we can represent all observables as matrices in an *N* dimensional Hilbert space. In the present case we have that the Hamiltonian is $H\left(t\right)=\sum_{i,j=1}^{N}h_{i,j}\left(t\right)E_{i,j}$ and the density matrix is $\rho \left(t\right)=\sum_{i,j=1}^{N}d_{i,j}\left(t\right)E_{i,j}$ . Note that for the hydrogen atom problem both *i* and *j* are triplets of quantum numbers so that the coefficient *d*_*i*,*j*_(*t*) is actually the coherence ${c_{l^{\prime },m^{\prime }}^{n^{\prime }}}^{*}\left(t\right)c_{l,m}^{n}\left(t\right)$ as mentioned above, e.g., eq 10

Inserting these expressions of ρ and **H** into the Liouville equation, and taking advantage of the commutation relations characteristic of the Gelfand set and their linear independence leads to a set of *N* ^2^ simultaneous first order linear differential equations for the elements of the density matrix,

13 $$\left(i\partial d_{i,j}/\partial t\right)=\sum_{ij,kl}{L\left(t\right)}_{ij,kl}d_{k,l}$$

Due to the sparsity of the Hamiltonian matrix (for many *n*,*m*,*h*_*n*,*m*_(*t*) = 0 ∀ *t*) there will be far fewer than *N* ^2^ simultaneous equations. There are two algebraic-based numerical schemes that are complementary, for integrating matrix differential equations such as eq 12. The approach of Magnus^30,31^ where the evolution is exponential in the sum of the generators, and the scheme of Wei and Norman^32–36^ where the evolution is a product of exponentials, each exponent being one generator. Both approaches have been extensively used in the literature. We have tended to use the scheme of Wei and Norman.^34,37^ When the size, *N*, of the Hilbert space is not large, a direct numerical integration of the *N* coupled first order differential equations works well.

Of course, a strictly exact numerical solution requires taking *N* → ∞ while we will work with a finite value of *N*. But computer arithmetic has a finite accuracy and as long as we are within that limit, typically below 1 in 10^–16^, a finite value of *N* can be accurate in a pragmatic sense.

This algebraic approach could be used to do the dynamics, and this approach could be done very efficiently if a closed algebra were found which could exploit the structure of the Hamiltonian. An additional motivation for an algebraic approach is the close and intimate connection with the maximal entropy formalism, dynamical symmetries^38,39^ and surprisal analysis.^40,41^

## Results: Electronic Stereodynamics

### The Dynamics for Asymmetry

We examine a hydrogen atom, initially in its ground 1s state, perturbed by a series of weak, one-photon linearly and circularly polarized optical pulses. Before looking at a chiral final state, we will first look at the excitation of a state which is merely asymmetric in a single plane of reflection. The system begins in the ground state and is excited with three optical perturbations. The first pulse is resonant with the transition between the 1s and 2p_1_ states, ω_1_ = *E*_2_ – *E*_1_. A second pulse moves population from the initial 1s state to the 3p_1_ state, and is resonant with this transition, ω_2_ = *E*_3_ – *E*_1_. The third pulse, exciting from the 2p_1_ to the 3d_1_ states, is resonant with this transition, ω_3_ = *E*_3_ – *E*_2_. The schematic for this process is presented in Figure 3 panel a.

**Figure 3:**
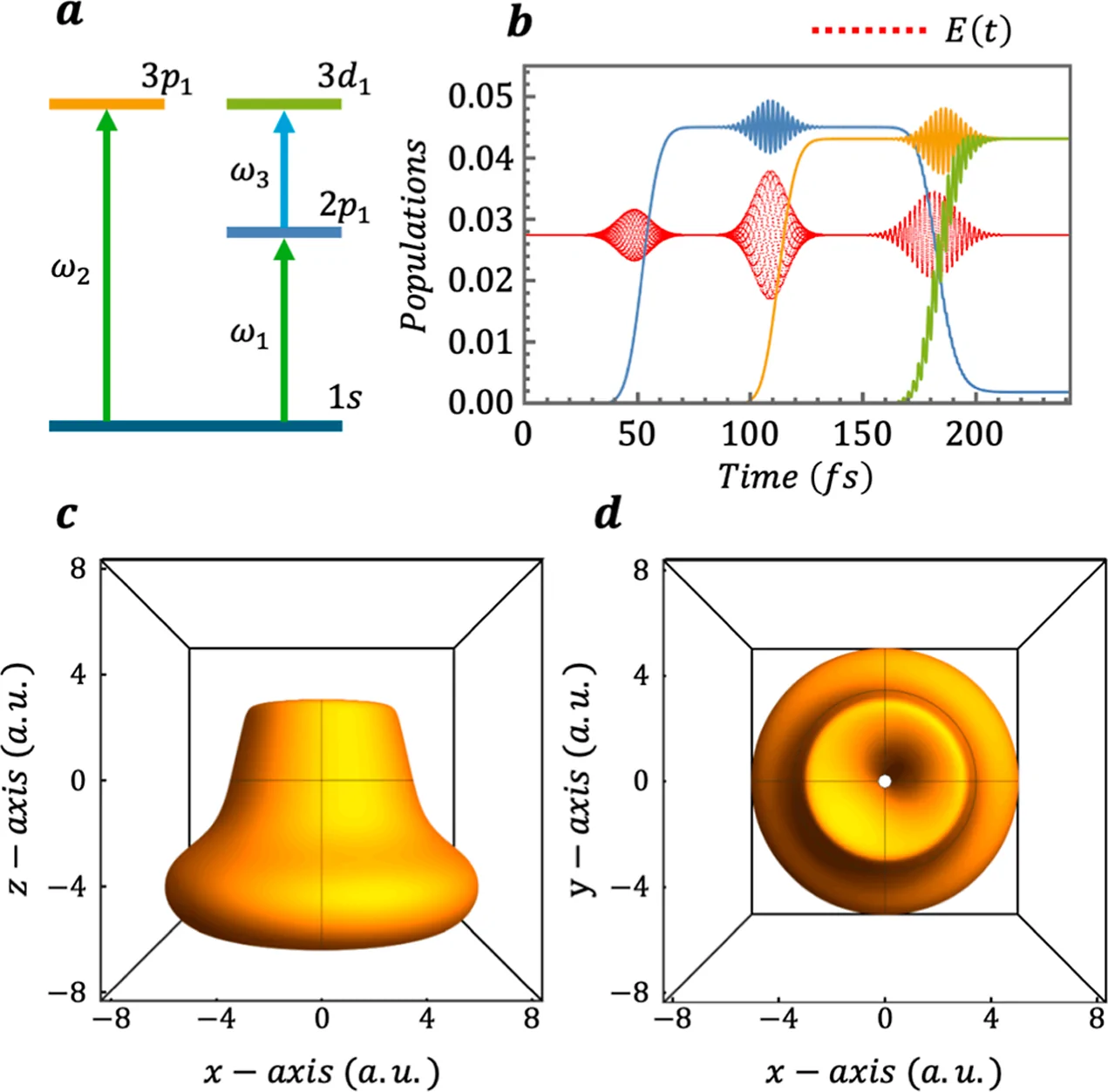
(a) Schematic of the excitation of the ground state using the first pulse setup, eq 14. (b) Dynamics of the 2p_1_, 3p_1_ and 3d_1_ populations caused by this pulse configuration. The coloring of the populations on this panel corresponds to the labeled levels on panel a: the blue line is the 2p_1_ population; the orange line is the 3p_1_ population; and the green line is the 3d_1_ population. The red dashed line on the figure shows the positions and widths of the pulse sequence exciting the system. The bottom panels show the isosurface of eq 17, the infinitesimally thin shell on which the density is ρ′(*r*,τ) = 10^–5^,where τ is a time after the pulses have ceased, τ = *t*_3_ + 45fs. (c) faces the *x*–*z* plane and (d) faces the *x*–*y* plane.

The asymmetry inducing pulse setup, which is similar but not identical to the pulse setup in,^16^ eqs 2 to 4, is made up of three pulses. Pulses 1 and 2 are circularly polarized in the *x*–*y* plane and pulse 3 is linearly polarized along the *z*-axis. The pulses are identified with subscripts

14 $$\begin{array}{l} E_{x}\left(t\right)=g_{1}\left(t\right)\cos\left(\omega_{1}t\right)+g_{2}\left(t\right)\cos\left(\omega_{2}t\right)\\ E_{y}\left(t\right)=g_{1}\left(t\right)\sin\left(\omega_{1}t\right)+g_{2}\left(t\right)\sin\left(\omega_{2}t\right)\\ E_{z}\left(t\right)=g_{3}\left(t\right)\cos\left(\omega_{3}t\right) \end{array}$$

*g*_*i*_(*t*) is the pulse envelope taken here to be a gaussian

15 $$g_{i}\left(t\right)=E_{i}\exp\left[-\frac{{\left(t-t_{i}\right)}^{2}}{2{\sigma_{i}}^{2}}\right]$$

where σ_*i*_ is the pulse width and *t*_*i*_ is its temporal center. To simplify subsequent equations, the pulse strength, $E_{i}$ , is absorbed into *g*_*i*_(*t*). Note that with this definition, there is de facto a carrier envelope phase (CEP) coming from the fact that the max of the envelope does not coincide with the max of the electric field of the pulse.

Values of $E_{1}=5.4\times 10^{-4}a.u$ , $E_{2}=1.35\times 10^{-3}a.u$ and $E_{3}=1.29\times 10^{-3}a.u$ are chosen so as not to overly depopulate the ground state. Values of σ_1_ = σ_2_ = 7.5fs and σ_3_ = 10.0fs are broad enough that the pulses are only resonant with the specific state transitions. The time delays are sufficiently large that, despite their broad widths, the pulses do not overlap in the time domain. Relative to a start time of *t* = 0 well before the pulse sequence, the first pulse is centered at *t*_1_ = 50fs, the second pulse is centered at *t*_2_ = 110fs and the third is centred at *t*_3_ = 180fs.

Equation 12 is used to calculate the density matrix of the system after the pulse has completed, **ρ**(*t*). The ground state is depopulated by approximately 9%. Figure 3b shows the dynamics of the 2p_1_, 3p_1_ and 3d_1_ populations.

Because of the significant energy difference between the hydrogen ground state and higher excited states (*E*_2_ – *E*_1_ = 10.2 eV) the coherences between the ground and excited state created by the perturbation will decouple extremely quickly. Consequently, the ground and higher excited states can be treated as uncorrelated

16 $$\mathrm{\rho ̂}\left(t\right)\approx {\mathrm{\rho ̂}}_{G}\left(t\right)+{\mathrm{\rho ̂}}^{\prime }\left(t\right)$$

Where ${\mathrm{\rho ̂}}_{G}\left(t\right)={\left|c_{0,0}^{1}\left(t\right)\right|}^{2}|1,0,0\rangle \langle 1,0,0|$ and ${\mathrm{\rho ̂}}^{\prime }\left(t\right)$ is the higher order excited state populations and coherences. The density function of this latter term can be calculated in coordinate space as

17 $$\rho^{\prime }\left(r,t\right)=\left\langle r|{\mathrm{\rho ̂}}^{\prime }\left(t\right)|r\right\rangle$$

An isosurface is defined as usual as the envelope of the function where it has a specific value. In other words, the isosurface is the infinitesimally thin shell where the density is equal to a specified value. Figure 3 panels c and d show an isosurface of eq 17. The maximum value of the density, *max*[ρ′(**r**,τ)] = 5.9 × 10^–5^. To get a better sense of the shape of the density, the isosurface displayed in Figure 3 panels c and d is for a slightly lower value, of the same order of magnitude. The shell on which the density is ρ′(**r**,τ) = 10^–5^,where time τ long after the third pulse has ceased, τ = *t*_3_ + 45 fs. It can be seen by eye that, while this density function is invariant under reflection in the *x*–*z* and *y*–*z* planes, it is non-invariant under reflection in the *x*–*y* plane.

Using the S_1_ operators, this means that

$$\begin{array}{l} {\mathrm{\sigma ̂}}_{xy}\rho \left(r,\theta ,\phi ,\tau \right)\neq \rho \left(r,\theta ,\phi ,\tau \right)\\ {\mathrm{\sigma ̂}}_{xz}\rho \left(r,\theta ,\phi ,\tau \right)=\rho \left(r,\theta ,\phi ,\tau \right)\\ {\mathrm{\sigma ̂}}_{yz}\rho \left(r,\theta ,\phi ,\tau \right)=\rho \left(r,\theta ,\phi ,\tau \right) \end{array}$$

### The Dynamics for Chirality

The second pulse setup, designed to bring the system to a chiral superposition of hydrogenic states is made up of 4 pulses. Pulse 1 is linearly polarized along the *y*-axis, pulse 2 is linearly polarized along the *x*-axis, and pulses 3 and 4 are linearly polarized along the *z* axis.

$$\begin{array}{l} E_{x}\left(t\right)=g_{2}\left(t\right)\cos\left(\omega_{2}t\right)\\ E_{y}\left(t\right)=g_{1}\left(t\right)\cos\left(\omega_{1}t+\varphi \right)\\ E_{z}\left(t\right)=g_{3}\left(t\right)\cos\left(\omega_{3}t\right)+g_{4}\left(t\right)\cos\left(\omega_{4}t\right) \end{array}$$

As before, the frequency of the first pulse is resonant with the transition of from *n* = 1 to 2, ω_1_ = *E*_2_ – *E*_1_, which moves population from initial 1s state to the 2p_–1_ and 2p_1_ states. The second pulse is resonant with the transition from *n* = 1 to 3, ω_2_ = *E*_3_ – *E*_1_, which moves population from the initial 1s state to the 3p_–1_ and 3p_1_ states. And the third pulse is resonant with the transition from *n* = 2 to 3, ω_3_ = *E*_3_ – *E*_2_, which moves population from 2p_–1_ to 3d_–1_ and from 2p_1_ to 3d_1_. In this setup, there is a fourth pulse which is resonant with the transition from the *n* = 3 to 4, ω_4_ = *E*_4_ – *E*_3_, which moves population from 3p_–1_ → 4d_–1_, 3p_1_ → 4d_1_, 3d_–1_ → 4f_–1_ and 3d_1_ → 4f_1_. The schematic for this setup is given in Figure 4 panel a.

**Figure 4:**
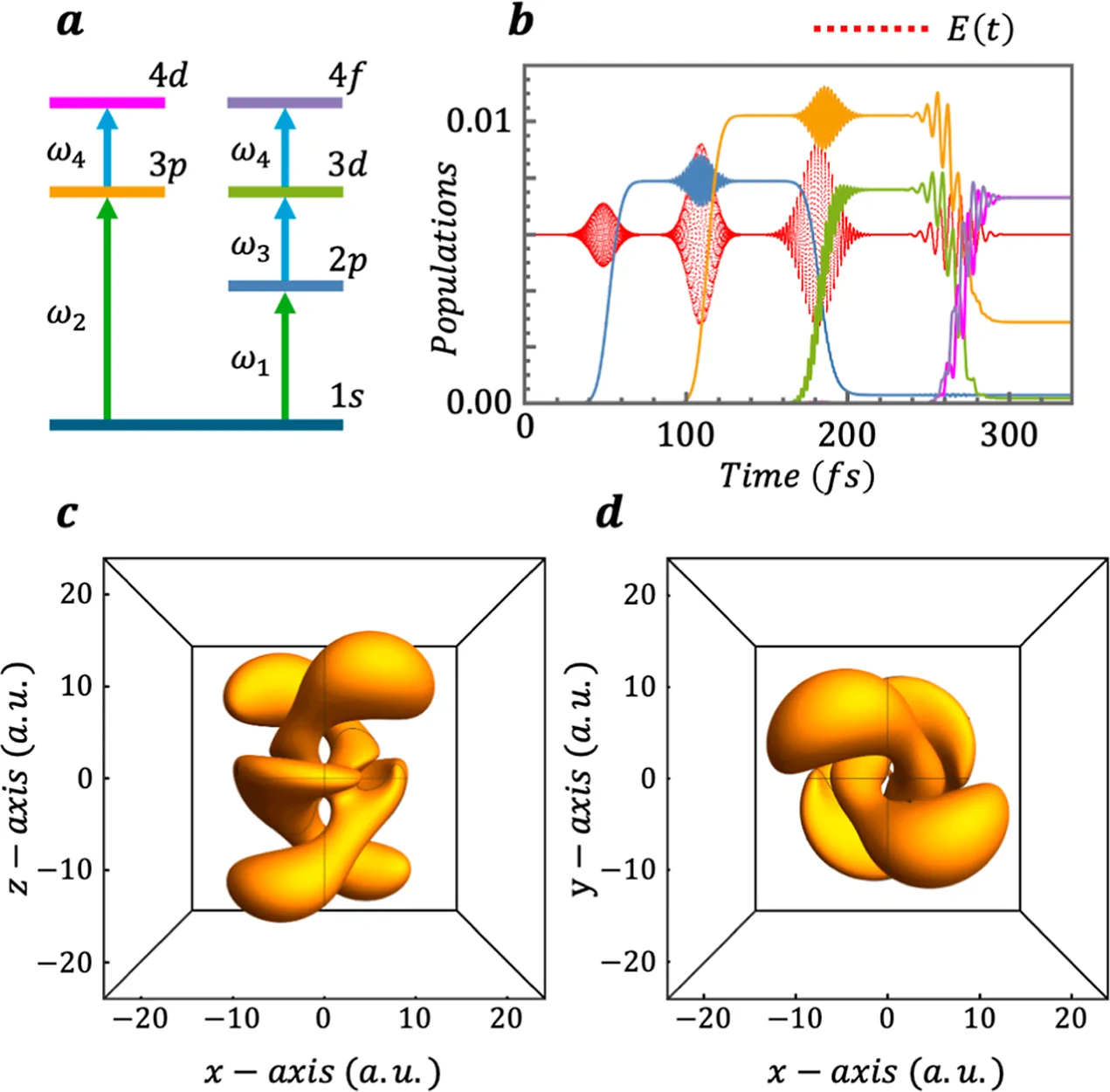
(a) Schematic of the excitation of the ground state using the chiral pulse setup given in (16). (b) Dynamics of the 2p_1_, 3p_1_, 3d_1_, 4d_1_ and 4f_1_ populations caused by this pulse configuration. The coloring of the populations on this panel corresponds to the labeled levels on panel a: as on Figure 3, the blue line is the 2p_1_ population; the orange line is the 3p_1_ population; and the green line is the 3d_1_ population; additionally, the pink line is the 4d_1_ population and the violet line is the 4f_1_ population. The red dashed line on the figure shows the positions and widths of the pulse sequence exciting the system. The bottom panels show the isosurface, the infinitesimally thin shell at which ρ′(*r*,τ) = 10^–6^,where τ is a time after the pulses have ceased, τ = *t*_4_ + 72.5fs. (c) faces the *x*–*z* plane and (d) faces the *x*–*y* plane.

The pulse widths are σ_1_ = σ_2_ = 7.5fs and σ_3_ = σ_4_ = 10.0fs. The pulses are centered sufficiently far apart that, despite their broad widths, they do not overlap. The first pulse is centered at *t*_1_ = 50fs, the second pulse at *t*_2_ = 110fs, the third pulse at *t*_3_ = 180fs and the fourth pulse at *t*_4_ = 265fs. The following pulse strengths are used, $E_{1}=4.5\times 10^{-4}$ , $E_{2}=1.29\times 10^{-3}$ $E_{3}=1.3\times 10^{-3}$ and $E_{4}=6.0\times 10^{-4}$ . The phase added to the first pulse is $\varphi =-\frac{3\pi }{2}$ . As before, the φ is in addition to the CEP de facto present.

This pulse setup depopulates the ground state by approximately 5%. The dynamics of the 2p_1_, 3p_1_, 3d_1_, 4d_1_ and 4f_1_ populations produced by this pulse configuration are given in Figure 4 panel b. The dynamics of the 1s and *m* = −1 states are given in Section S4 of the Supporting Information.

As before, the ground and excited states are taken to be decoupled. The maximum value of the density, *max*[ρ′(**r**,τ)] = 1.2 × 10^–5^. To get a better sense of the shape of the density, the isosurface displayed in Figure 4 panels c and d is for a slightly lower value, of the same order of magnitude. The infinitesimally thin shell on which the density is ρ′(**r**,τ) = 10^–6^, where τ is a time after the pulses have ceased, τ = *t*_4_ + 72.5 fs, is given on Figure 4 panels c and d.

The shape of the density is two interwoven twists, which like two intertwined corkscrews, is evidently chiral. An explicit comparison of ρ′ to *S*_*n*_ρ′ is given in Section S5 of the Supporting Information for *n* = 1,2,4.

It is important to note that the isosurfaces presented in this work, which show the qualitative asymmetry and chirality of the system after the perturbations, on Figures 3 and 4 respectively, are taken at specific points in time. However, there is in fact a time dependence to the shape of the densities and, by extension, to their asymmetry and chirality, even after the pulse sequences have ended.

In the systems presented in this work, this time dependence comes from the coherences between states with different values of the *n* quantum number. As such, the nature and extremity of the time dependent variation one observes in the symmetries is dependent upon the ratios of the populations of different states, as well as the relative phases of the coherences.

The time dependence of the asymmetry and chirality is a very interesting area of study, however it is sufficiently large and significant as to merit discussion and publication in a follow up paper. Where this paper has qualitatively explored the symmetry and chirality of systems, following from the algebraic approach, our follow up will be more quantitative. And, where this paper has presented the dynamics to induce asymmetry and chirality, its sequel will look at the dynamics of these properties.

## Conclusion

The algebraic definition of chirality is complementary to the familiar geometrical definition. The distinction between these two definitions is coterminous with the differences in the notions of symmetry conceived of by Sophus Lie and Felix Klein.^42^ As is well recorded, the two met in Paris in 1869 where they divided the world of symmetry between them with Klein pursuing the geometric and Lie the algebraic approach. Here we followed Lie, but with high attention to the work of Klein whose views dominated many areas and also influenced the science of chirality. The algebraic approach provides the opportunity for a conceptual expansion from classical to quantum chirality.

We have provided examples by which asymmetry and chirality, both time dependent and static, can be characterized and induced in symmetric atoms by chiral perturbations. And we have provided group theoretic confirmation of these properties, derived from the algebraic definitions of asymmetry and chirality.

In this work we have only investigated classical chirality in localized electronic stereodynamics. However, the algebraic approach has established the framework in which one could fruitfully investigate the quantum chirality of non-localized densities.

The twisted electronic density shown in Figure 4 reflects the interference between rather low-lying states of hydrogen. In the very early days of attoscience^43^ many of the available pulses were in a higher energy region. They could therefore excite electrons that are more bound than the valence shell. It might be interesting to return to this energy range and prepare a chiral electronic deeper lying states.^44^ Would it transfer the chirality to the valence electrons or to the nuclear frame? Or both?

## Supplementary Material



## Notes

Which contains five sections. Section 1: Glossary: An algebraic view of symmetry Section 2: Proof that rotations are shift operators about the angle of the axis Section 3: Derivation of the generators of reflection Section 4: The relatively sparse dipole matrices in the Hilbert space spanned by the electronic hydrogen states Section 5: Dynamics of chiral perturbation Section 6: Chiral density, acted upon by the operators of S_1_, S_2_, and S_4_ (DOCX)
