# Supplementary material for: Chirality Inherent and Induced: An Algebraic Point of View
Source: J Am Chem Soc. 2026 Sep 29;148(40):43535–44. doi: 10.1021/jacs.6c13874 (PMC13674270; doi:10.1021/jacs.6c13874)
Supplement: Supplementary file 1 [file ja6c13874_si_001.docx]

Supplementary Information: Chirality Inherent and Induced: An Algebraic Point of View

*James R. Hamilton^1^, Francoise Remacle^1,2^, Raphael D. Levine^1,3,4,^ ^[1]^**

^1^Institute of Chemistry, The Hebrew University of Jerusalem, Jerusalem 91904, Israel

^2^Theoretical Physical Chemistry, UR MOLSYS, University of Liege, B4000, Belgium

^3^ Department of Molecular and Medical Pharmacology, David Geffen School of Medicine, University of California, Los Angeles, CA 90095, USA

^4^ Department of Chemistry and Biochemistry, University of California, Los Angeles, CA, 90095, USA

**Contents**

**S1**: Glossary: An algebraic view of symmetry

**S2**: Proof that rotations are shift operators about the angle of the axis

**S3**: Derivation of the generators of reflection

**S4**: The relatively sparse dipole matrices in the Hilbert space spanned by the electronic hydrogen states

**S5:** Dynamics of chiral perturbation

**S6**: Chiral density, acted upon by the operators of $S_{1}, S_{2}$ and $S_{4}$

**Section** **S1: Glossary: An Algebraic View of Symmetry**

*Symmetry groups*. We know of two types of symmetry groups, both of which satisfy the axioms of group theory. The first type is the discrete symmetries which includes rotations by finite amounts, for example, 60 degrees or reflections by 180 degrees in a plane. The second type are the continuous symmetries, eg., rotation by any angle, including infinitely small increments. Some of these, such as rotation in a 2D plane or a 3D space are well known.

*Symmetry algebras*. A special property of a continuous symmetry is that it is associated with an algebra as revealed in brilliant and exceptional detail by Sophus Lie. So, the algebra is now known as a Lie algebra.

*A Lie algebra*. A set of linear operators that is said to be ‘closed’ under the binary operation of a commutation. This is a key property because it replaces a ‘product’ of two operators, namely the commutator, by a term linear in the operators. For future use we will use a double index for the operators of a Lie group so that $\left[ E_{ij}, E_{kl} \right]=E_{ij} E_{kl}-E_{kl}E_{ij}=\sum_{mn} c_{ijkl}^{mn}E_{mn}$here $c_{ijkl}^{mn}$ is a numerical coefficient. The members of the Lie algebra are often known as the generators because they generate the displacement of a system from its initial state.

*Computational quantum chemistry*. When we use an orthogonal basis set, say $\left\{ |\left. i \right\rangle\right\}$ we have a Lie algebra, $E_{ij}=|\left. i \right\rangle\left\langle j| \right..$ Please note that any matrix observable in this basis is a linear combination of the matrix representation of the generators. The generators $E_{ij}=|\left. i \right\rangle\left\langle j| \right.$ are not Hermitian but $\left( E_{ij}+E_{ij}^{\dagger} \right)/2$ and $\left( E_{ij}-E_{ij}^{\dagger} \right)/{2i}$are.

*Constructing a Lie algebra*. The mathematics leading from a Lie group to a Lie algebra is not always easy, and it can be a significant entry barrier for newcomers. Recently people, us included, prefer to start with the algebra. This has many advantages but at least one drawback: it creates a break with the familiar approach to symmetry.

*Constructing a Lie group.* If the Hamiltonian is a combination of generators of a Lie algebra, a corresponding Lie group is what people in dynamics call the evolution operator. It generates the motion of the system in time. This is relatively easy to show in principle if the Hilbert space is finite dimensional. We have recently published several examples of applications of such an approach including references to others.

Take home partial summary. *The generators of a Lie algebra of a dynamical system describe those transition that are allowed coherently between states*. As such, it is possible to specify the

algebra on physical grounds. This allows for an efficient algebraic treatment of the dynamics. Most transitions proceed sequentially so that corresponding generators need not be included. There is no absolute harm if they are, it only complicates the algebra quadratically. So better to avoid.

**Section** **S2: Proof that rotations are shift operators about the angle of the axis**

$\hat{R}_{j}=\exp\left( -i\Delta\vartheta\hat{J}_{j} \right)$ is a Taylor operator which moves the argument of a function $f\left( \vartheta\right)$ by $\Delta\vartheta$

For simplicity of notation, using $\omega\equiv\Delta\vartheta,$

$$\begin{aligned} \hat{R}_{j}f\left( \vartheta\right)=f\left( \vartheta-\omega\right) \#\left( S1 \right) \end{aligned}$$

Using the differential expression of $\hat{J}_{j}=-i\frac{\partial}{\partial\vartheta}$, then $\hat{R}_{i}\left( \omega\right)=\exp\left[ -\omega\frac{\partial}{\partial\vartheta} \right]$. Using the power series expansion of $f\left( \vartheta\right)=\sum_{k} f_{k}\vartheta^{k}$. Then equation $\left( S1 \right)$ becomes

$$\begin{aligned} \hat{R}_{i}\left( \vartheta\right)f\left( \vartheta\right)=\sum_{k} f_{k}\hat{R}_{i}\left( \vartheta\right)\vartheta^{k} \boldsymbol{\#}\left( S2 \right) \end{aligned}$$

One can look at how the rotation acts on each polynomial $\vartheta^{k}$ by doing the Taylor expansion of the exponential

$$\begin{aligned} \hat{R}_{i}\left( \omega\right)\vartheta^{k}=\exp\left[ -\omega\frac{\partial}{\partial\vartheta} \right]\vartheta^{k}=\left( 1-\omega\frac{\partial}{\partial\vartheta}+\frac{\left( -\omega\right)^{2}}{2!}\frac{\partial^{2}}{{\partial\vartheta}^{2}}+\frac{\left( -\omega\right)^{3}}{3!}\frac{\partial^{3}}{{\partial\vartheta}^{3}}+\frac{\left( -\omega\right)^{4}}{4!}\frac{\partial^{4}}{{\partial\vartheta}^{4}}\ldots\right)\vartheta^{k} \boldsymbol{\#}\left( S3 \right) \end{aligned}$$

By evaluating this expression for specific values of $k$: $\hat{R}_{i}\left( \omega\right)\vartheta=\left( \vartheta-\omega\right)$; $\hat{R}_{i}\left( \omega\right)\vartheta^{2}=\left( \vartheta-\omega\right)^{2}$; $\hat{R}_{i}\left( \omega\right)\vartheta^{3}=\left( \vartheta-\omega\right)^{3}$ one can simplify equation $\left( S3 \right)$ to

$\hat{R}_{i}\left( \omega\right)\vartheta^{k}=\left( \vartheta-\omega\right)^{k}$

Substituting this into equation $\left( S2 \right)$ yields

$\hat{R}_{i}\left( \vartheta\right)f\left( \vartheta\right)=\sum_{k} f_{k}\hat{R}_{i}\left( \vartheta\right)\vartheta^{k}=\sum_{k} f_{k}\left( \vartheta-\omega\right)^{k}=f\left( \vartheta-\omega\right)$

In this way, equation $\left( S1 \right)$ is recovered

**Section** **S3: Derivation of the generators of reflection**

The continuous reflection operator in the $x-z$ plane is

$\begin{aligned} \hat{\sigma}_{xz}\left( \vartheta\right)=\exp\left[ -i\vartheta\hat{\varpi}_{xz} \right] \#\left( S4 \right) \end{aligned}$

In spherical polar coordinates, the generator of reflection in this plane is

$\begin{aligned} \hat{\varpi}_{xz}\equiv\phi\frac{\partial}{\partial\phi}, \#\left( S5 \right) \end{aligned}$

The constriction of this definition is that, essentially, $\hat{\varpi}_{xz}$ a scaling operator. $\exp\left[ k\phi\frac{\partial}{\partial\phi} \right]$ scales a by $e^{k}$.

$\exp\left[ k\phi\frac{\partial}{\partial\phi} \right]f\left( \phi\right)=f\left( e^{k}\phi\right)$

So, when $k=\pi,$ as in the discrete normal reflection $\hat{\sigma}_{xz}\left( \pi\right)=\exp\left[ -i\pi\hat{\varpi}_{xz} \right]$, this function scales by $e^{-i\pi}=-1$. Therefore

$$\begin{aligned} \exp\left[ -i\pi\phi\frac{\partial}{\partial\phi} \right]f\left( r,\theta,\phi\right)=f\left( r,\theta,e^{-i\pi}\phi\right)=f\left( r,\theta,-\phi\right) \#\left( S6 \right) \end{aligned}$$

This can be unpacked further by looking at specific $f\left( \phi\right)$. First, using $f\left( \phi\right)=\phi^{k}$

$\hat{\varpi}_{xz}\phi^{k}=\phi k\phi^{k-1}=k\phi^{k}$

${\hat{\varpi}_{xz}}^{2}\phi^{k}=k^{2}\phi^{k}$

Therefore

$$\begin{aligned} {\hat{\varpi}_{xz}}^{n}\phi^{k}=k^{n}\phi^{k} \#\left( S7 \right) \end{aligned}$$

Doing a Taylor expansion of the discrete reflection operator $\hat{\sigma}_{xz}\left( \pi\right)=\exp\left[ -i\pi\hat{\varpi}_{xz} \right]$

$\exp\left[ -i\pi\hat{\varpi}_{xz} \right]\phi^{k}=\left( \sum_{n=0}^{\infty} \frac{\left( -i\pi\right)^{n}{\hat{\varpi}_{xz}}^{n}}{n!} \right)\phi^{k}=\left( \sum_{n=0}^{\infty} \frac{\left( -i\pi\right)^{n}{\hat{\varpi}_{xz}}^{n}\phi^{k}}{n!} \right)$

Substituting equation $\left( S7 \right)$ into this expansion yields

$\exp\left[ -i\pi\hat{\varpi}_{xz} \right]\phi^{k}=\left( \sum_{n=0}^{\infty} \frac{\left( -i\pi\right)^{n}k^{n}\phi^{k}}{n!} \right)=\left( \sum_{n=0}^{\infty} \frac{\left( -i\pi\right)^{n}k^{n}}{n!} \right)\phi^{k}=e^{-i\pi k}\phi^{k}$

$\exp\left[ -i\pi\hat{\varpi}_{xz} \right]\phi^{k}=e^{-i\pi k}\phi^{k}=\left( e^{-i\pi}\phi\right)^{k}=\left( -\phi\right)^{k}$

$f\left( \phi\right)$ that can be expanded in a power series

$f\left( \phi\right)={\sum_{k} f_{k}\phi}^{k}$

$\exp\left[ -i\pi\hat{\omega}_{xz} \right]f\left( \phi\right) = exp\left[ -i\pi\hat{\omega}_{xz} \right]{\sum_{k} f_{k}\phi}^{k}$

$\exp\left[ -i\pi\hat{\omega}_{xz} \right]f\left( \phi\right)={\sum_{k} f_{k}\exp\left[ -i\pi\hat{\omega}_{xz} \right]\phi}^{k}$

$\exp\left[ -i\pi\hat{\omega}_{xz} \right]f\left( \phi\right)=\sum_{k} f_{k}\left( \sum_{n=0}^{\infty} \frac{\left( -i\pi\right)^{n}k^{n}}{n!} \right)\phi^{k}$

$\exp\left[ -i\pi\hat{\omega}_{xz} \right]f\left( \phi\right)=\sum_{k} f_{k}exp\left[ -i\pi k \right]\phi^{k}$

$\exp\left[ -i\pi\hat{\omega}_{xz} \right]f\left( \phi\right)=\sum_{k} f_{k} \left( -\phi\right)^{k}$

$$\exp\left[ -i\pi\hat{\omega}_{xz} \right]f\left( \phi\right)=f\left( -\phi\right)$$

To make this fully explicit, using $f\left( \phi\right)=\exp\left[ -im\phi\right]$

$\exp\left[ i\pi\hat{\varpi}_{xz} \right]\exp\left[ im\phi\right]=\exp\left[ i\pi\hat{\varpi}_{xz} \right]\left( \phi^{0}+im\phi^{1}+\frac{\left( im \right)^{2}}{2!}\phi^{2}+\frac{\left( im \right)^{3}}{3!}\phi^{3}+\frac{\left( im \right)^{4}}{4!}\phi^{4}\ldots\right)$

$\exp\left[ i\pi\hat{\varpi}_{xz} \right]\exp\left[ im\phi\right]=\left( \exp\left[ i\pi\hat{\varpi}_{xz} \right]\phi^{0}+im\exp\left[ i\pi\hat{\varpi}_{xz} \right]\phi^{1}+\frac{\left( im \right)^{2}}{2!}\exp\left[ i\pi\hat{\varpi}_{xz} \right]\phi^{2}+\frac{\left( im \right)^{3}}{3!}\exp\left[ i\pi\hat{\varpi}_{xz} \right]\phi^{3}+\frac{\left( im \right)^{4}}{4!}\exp\left[ i\pi\hat{\varpi}_{xz} \right]\phi^{4}\ldots\right)$

$\exp\left[ i\pi\hat{\varpi}_{xz} \right]\exp\left[ im\phi\right]=\left( \left( -\phi\right)^{0}+im\left( -\phi\right)^{1}+\frac{\left( im \right)^{2}}{2!}\left( -\phi\right)^{2}+\frac{\left( im \right)^{3}}{3!}\left( -\phi\right)^{3}+\frac{\left( im \right)^{4}}{4!}\left( -\phi\right)^{4}\ldots\right)$

$\exp\left[ i\pi\hat{\varpi}_{xz} \right]\exp\left[ im\phi\right]=\left( 1-im\phi+\frac{\left( im \right)^{2}}{2!}\phi-\frac{\left( im \right)^{3}}{3!}\phi+\frac{\left( im \right)^{4}}{4!}\phi\ldots\right)$

Therefore

$$\exp\left[ i\pi\hat{\varpi}_{xz} \right]\exp\left[ im\phi\right]=\exp\left[ -im\phi\right]$$

This confirms equations $\left( S4 \right)$, $\left( S5 \right)$, $\left( S6 \right)$ and $\left( S7 \right)$.

**Section** **S4: The relatively sparse dipole matrices in the Hilbert space spanned by the electronic hydrogen states**

Figures S1 and S2 show the matrix plots of the **Z** and **X** dipole matrices respectively. The dipole matrices being represented are defined in the Hilbert space of the states of the Hydrogen atom, $\left. \left| n,l,m \right. \right\rangle$, for $n=1$ to $5$. They are therefore $55\times55$ matrices. In the matrix plots, the top-most, left-most elements are $\left\langle\left. 1,0,0 \right| \right.x\left. \left| 1,0,0 \right. \right\rangle$ and $\left\langle\left. 1,0,0 \right| \right.z\left. \left| 1,0,0 \right. \right\rangle$.

Both **Z** and **X** are fully real. In the matrix plots a positive value element is coloured orange and a negative value element is coloured blue. The intensity of the colour signifies the magnitude of the element. Zero elements are white, and the propensity of these white elements in both figures shows the sparsity of the matrices. It is also notable that, as the quantum principle number $n$ increases, the corresponding parts of the matrices become ever sparser.

Figure S1: Matrix plot of the $\boldsymbol{Z}$ dipole matrix, defined in the Hilbert space spanned by the states of the Hydrogen atom, $\left. \left| n,l,m \right. \right\rangle$, for $n=1$ to $5$. The real symmetric matrix has elements $\left\langle\left. n',l',m' \right| \right.z\left. \left| n,l,m \right. \right\rangle$ arranged as submatrices of increasing n.

Figure S2: Matrix plot of the $\boldsymbol{X}$ dipole matrix, defined in the Hilbert space spanned by the states of the Hydrogen atom, $\left. \left| n,l,m \right. \right\rangle$, for $n=1$ to $5$

**Section** **S5: Dynamics of chiral perturbation**

Figure S3: The dynamics stimulated by the pulse configuration of equation (16) of the main text. a) The top panel shows the population of the ground, $1s$ state. b) the middle panel shows the populations of the $m=1$ states excited by the pulse: $2p_{1},$ yellow; $3p_{1}$, green; $3d_{1}$, purple; $4d_{1}$, brown; $4p_{1}$, blue. c) the bottom panel shows the populations of the $m=-1$ states excited by the pulse: $2p_{-1},$ yellow; $3p_{-1}$, green; $3d_{-1}$, purple; $4d_{-1}$, brown; $4p_{-1}$, blue. The red dashed line on each panel shows the pulse.

**Section** **S6: Chiral density, acted upon by the operators of** $\boldsymbol{S}_{\boldsymbol{1}}\mathbf{,}\boldsymbol{S}_{\boldsymbol{2}}$ **and** $\boldsymbol{S}_{\boldsymbol{4}}$

Table S1: The chiral density of figure 4 panel c of the main text subject to the improper rotations of $S_{1}, S_{2}$ and $S_{4}$. The density is facing in the $x-y$ plane. The top row shows the original, un-operated on density. The second row shows the density acted on by $S_{1}$ reflections. The third row shows the density acted on by the single $S_{2}$ improper rotation. The fourth row shows the density acted on by the three $S_{4}$ improper rotations.

|  | Original density, $\rho^{'}$ | | |
| --- | --- | --- | --- |
|  |  | | |
| $S_{1}$ | $\hat{\sigma}_{xy}\rho^{'}$ | $\hat{\sigma}_{xz}\rho^{'}$ | $\hat{\sigma}_{yz}\rho^{'}$ |
|  |  |  |  |
| $S_{2}$ | $R^{I}\left( 2 \right)\rho^{'}$ | | |
|  |  | | |
| $S_{4}$ | $R_{x}^{I}\left( 4 \right)\rho^{'}$ | $R_{y}^{I}\left( 4 \right)\rho^{'}$ | $R_{z}^{I}\left( 4 \right)\rho^{'}$ |
|  |  |  |  |

Table S2: The chiral density of figure 4 panel d of the main text subject to the improper rotations of $S_{1}, S_{2}$ and $S_{4}$. The density is facing in the $x-z$ plane. The rows are the same as table S6: first row, un-operated on density; second row, $S_{1}$ ; third row, $S_{2}$; fourth row, $S_{4}$.

|  | Original density, $\rho^{'}$ | | |
| --- | --- | --- | --- |
|  |  | | |
| $S_{1}$ | $\hat{\sigma}_{xy}\rho^{'}$ | $\hat{\sigma}_{xz}\rho^{'}$ | $\hat{\sigma}_{yz}\rho^{'}$ |
|  |  |  |  |
| $S_{2}$ | $R^{I}\left( 2 \right)\rho^{'}$ | | |
|  |  | | |
| $S_{4}$ | $R_{x}^{I}\left( 4 \right)\rho^{'}$ | $R_{y}^{I}\left( 4 \right)\rho^{'}$ | $R_{z}^{I}\left( 4 \right)\rho^{'}$ |
|  |  |  |  |

1. * Corresponding author. [Raphy@mail.huji.ac.il](mailto:Raphy@mail.huji.ac.il)
